# Supplementary material for: Cryo-EM structures of pannexin 1 and 3 reveal differences among pannexin isoforms
Source: Nat Commun. 2024 Apr 5;15:2942. doi: 10.1038/s41467-024-47142-6 (PMC10997603; doi:10.1038/s41467-024-47142-6)
Supplement: Supplementary file 1 — Supplementary Information [file 41467_2024_47142_MOESM1_ESM.pdf]

Supplementary information file

Cryo-EM structures of Pannexin 1 and 3 reveal differences among Pannexin isoforms

Nazia Hussain[1], Ashish Apotikar[1], Shabareesh Pidathala[1,3], Sourajit Mukherjee[1,4], Ananth Prasad Burada[1], Sujit Kumar Sikdar[1], Kutti R. Vinothkumar[2] & Aravind Penmatsa[1*]

Table of Contents

Supplementary Fig.1	Multiple sequence alignment of human PANX isoforms.
Supplementary Fig.2a	Cryo-EM processing workflow for $PANX1_{WT}$
Supplementary Fig.2b	Cryo-EM processing workflow for $PANX1_{WR/RD}$
Supplementary Fig.2c	Cryo-EM processing workflow for $PANX1_{R217H}$
Supplementary Fig.3	Cryo-EM processing workflow for PANX3
Supplementary Fig.4	Representative densities for PANX3
Supplementary Fig.5	Structural features of PANX isoforms
Supplementary Fig.6	Biochemical analysis of PANX3 and its mutants with a comparative assessment against PANX1 and PANX2 isoforms
Supplementary Fig.7	Biochemical analysis of PANX1 and its mutants
Supplementary Fig.8	Patch clamp and surface expression analysis for PANX1 and its mutants
Supplementary Fig.9	Size exclusion profiles of PANX1 and PANX3 constructs
Supplementary Fig.10	$PANX1_{R217H}$ alters ATP interactions and channel behaviour
Supplementary Fig.11	Raw data of patch clamp studies performed with PANX1
Supplementary Fig.12	Raw data from patch clamp studies involving PANX3
Supplementary Fig.13	Surface expression of wild-type PANX1 and the mutants
Supplementary Fig.14	Surface expression of wild-type PANX3 and the mutants
Supplementary Fig.15	SDS-PAGE profile for the constructs mentioned in the study
Supplementary Table 1	List of Primers used in the study along with their sequences

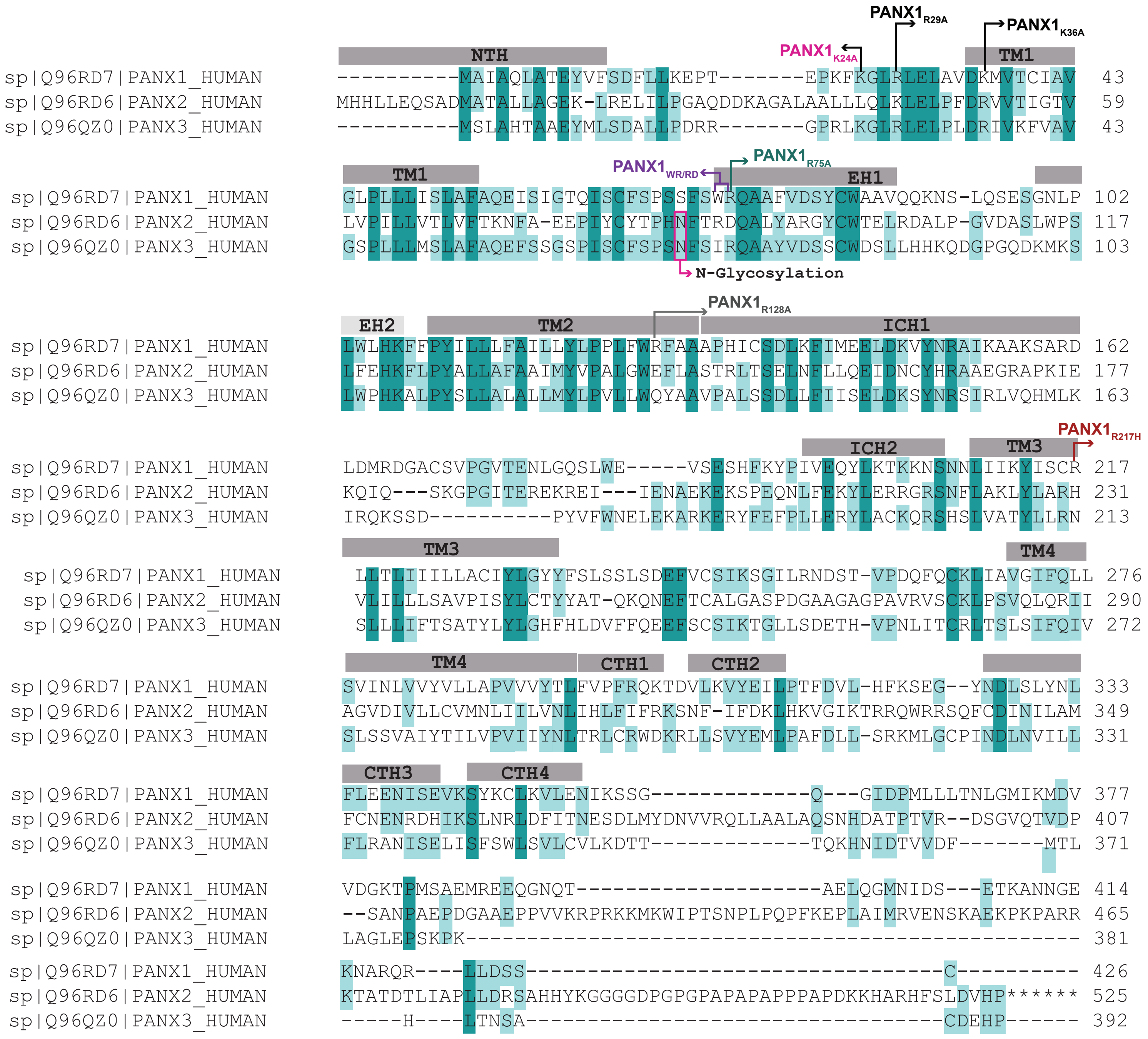

Supplementary Fig. 1: Multiple sequence alignment of human PANX isoforms. The mutants studied are marked. $PANX1_{WR/RD}$, represents a double mutant (W74R, R75D). $PANX1_{R217H}$ is a germline mutant, $PANX1_{R24A}$, $PANX1_{R29A}$ are N-terminus mutants, $PANX1_{K36A}$ is a TM1 mutant, $PANX1_{R75A}$ is a pore mutant, and $PANX1_{R128A}$ is a substitution at TM2. TM= Transmembrane, NTH=N-terminal Helix, ICH=Intracellular helix, CTH=C-terminal helix. N-glycosylation at position 86 in PANX2 and position 71 in PANX3 is also indicated.

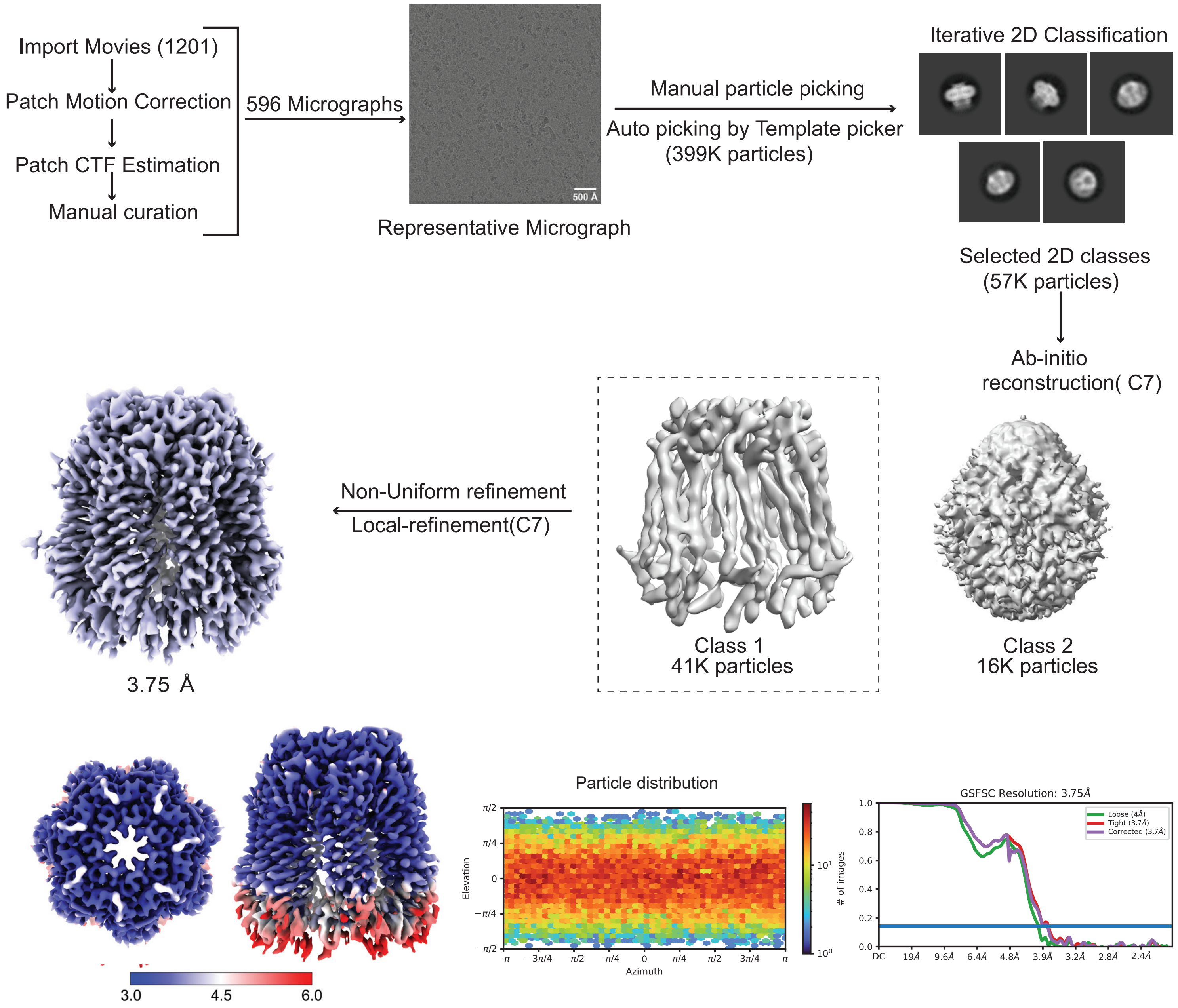

Supplementary Fig. 2a: Cryo-EM processing workflow for PANX1$_{WT}$. The processing was done in CryoSparc. C7 symmetry was applied for ab-initio and non-uniform refinement for the 3D reconstruction. A total of 41k particles were used for the final 3D reconstruction. Local resolution was estimated through CryoSparc local resolution estimation protocol.

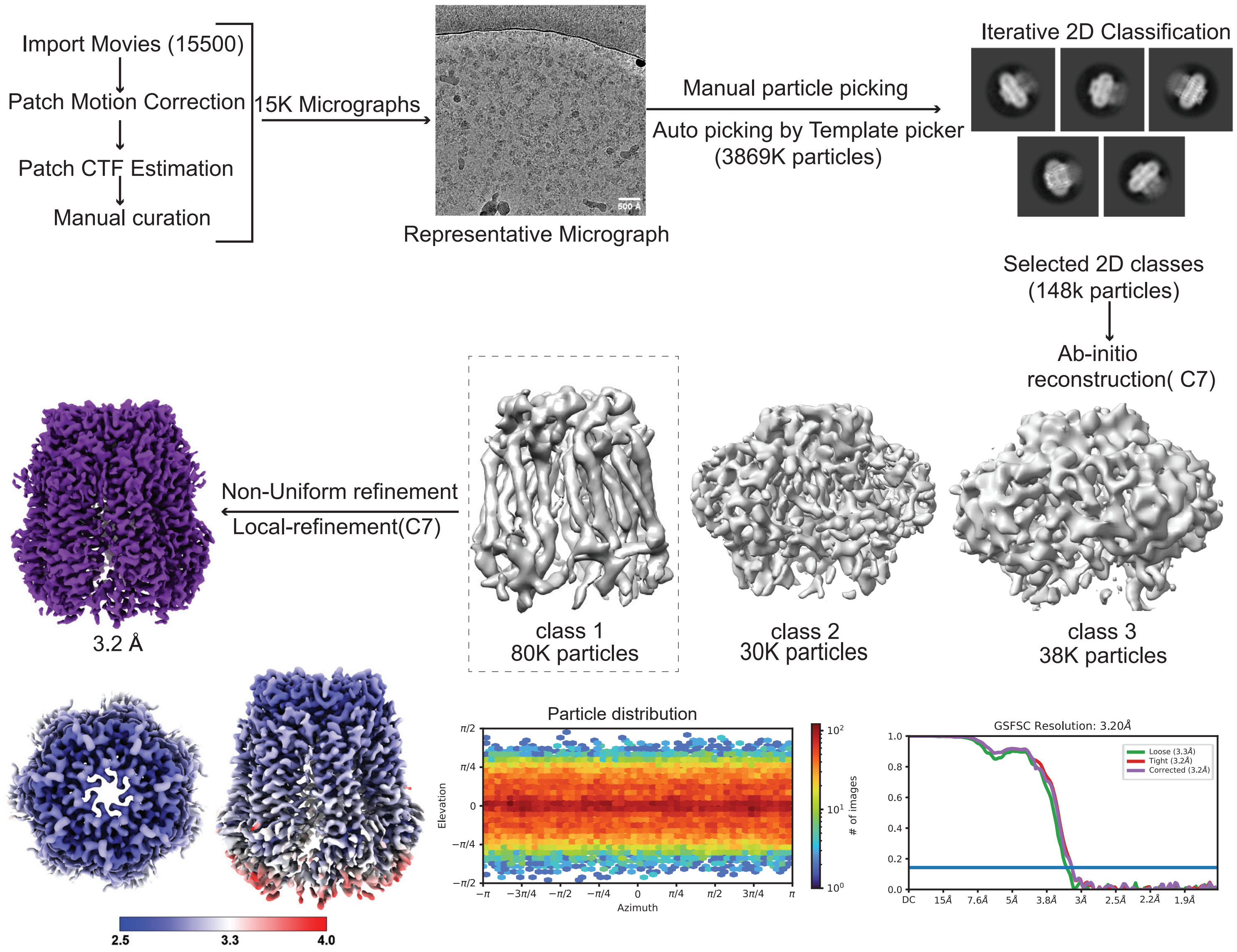

Supplementary Fig. 2b: Cryo-EM processing workflow for PANX1$_{WR/RD}$. All the processing was done in CryoSparc, C7 symmetry was applied for ab-initio and non-uniform refinement for the final reconstruction. A total of 80k particles were used for the final 3D reconstruction. Local resolution estimation was done through CryoSparc local resolution estimation. The data was collected at CM01, ESRF.

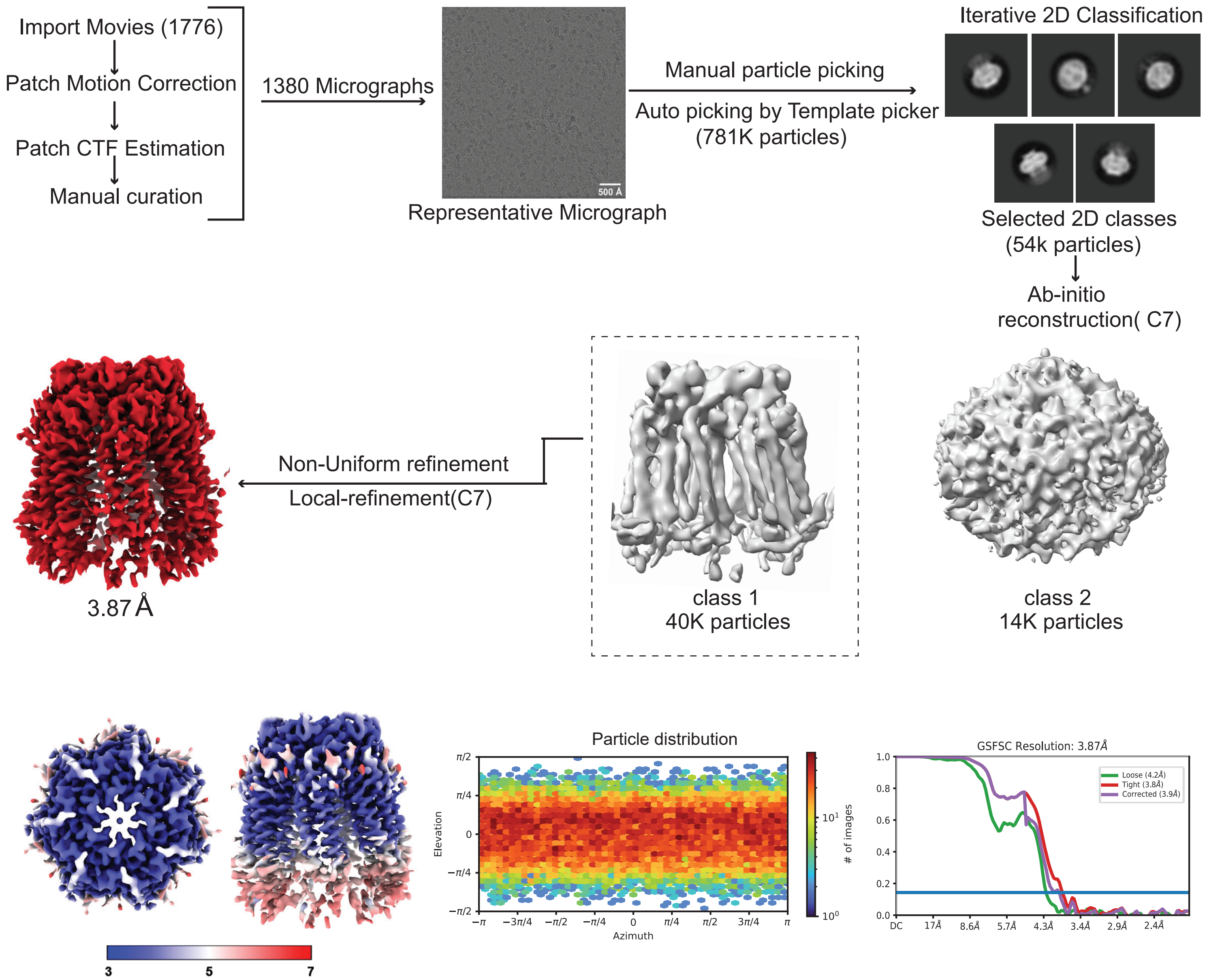

Supplementary Fig. 2c: Cryo-EM processing workflow for PANX1$_{R217H}$. All the processing was done in CryoSparc, C7 symmetry was applied for ab-initio and non-uniform refinement for the final reconstruction. A total of 40k particles were used for the final 3D reconstruction. The model used for refinement is represented by a dashed line. Local resolution estimation was done through CryoSparc local resolution estimation protocol.

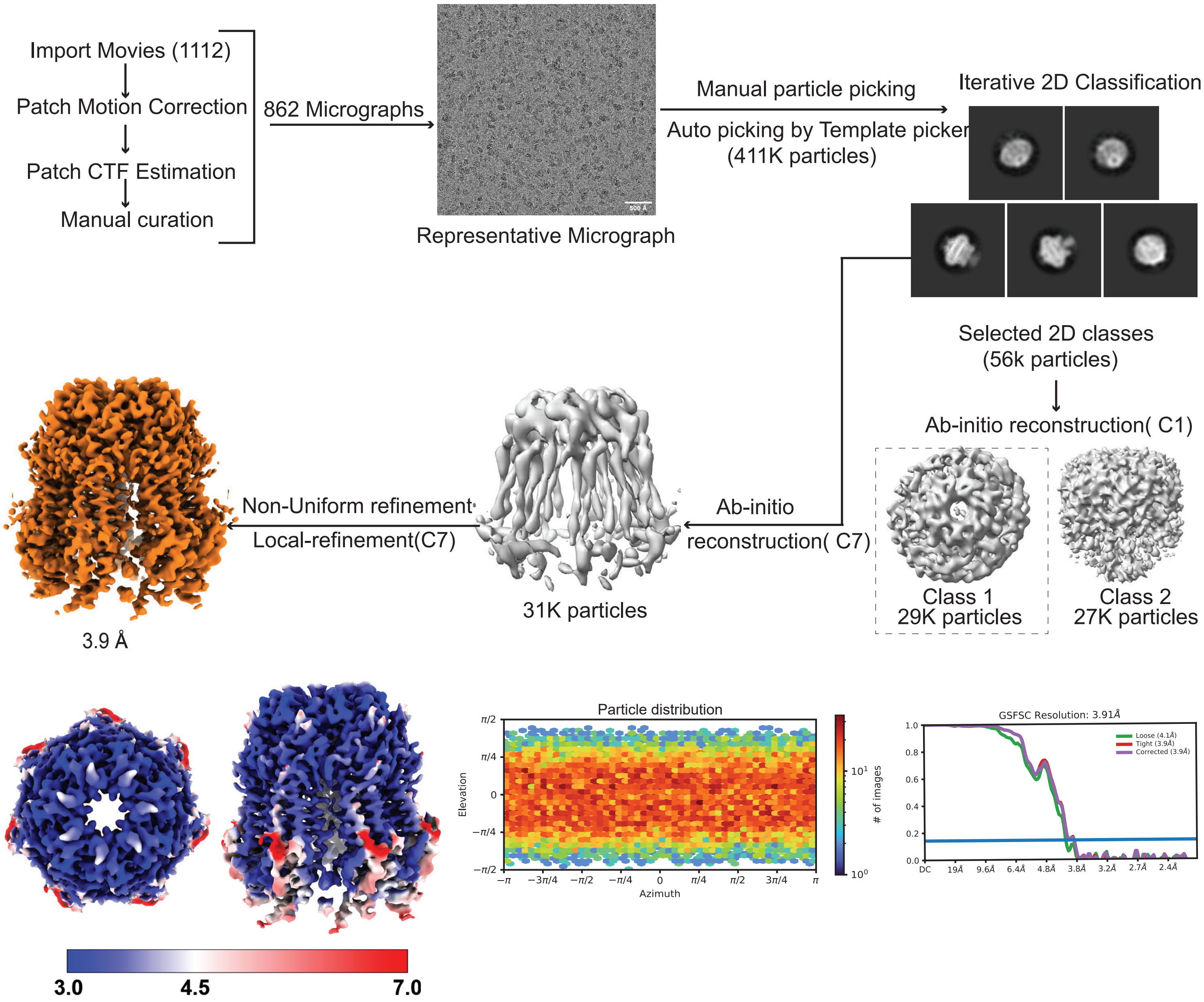

Supplementary Fig. 3: Cryo-EM processing workflow for PANX3. Initial ab-initio was done without symmetry. As seven subunits could be visualized in the ab-initio 3D reconstruction (Class 1), C7 symmetry was applied for ab-initio and non-uniform refinement for the final reconstruction. A total of 31k particles were used for the final 3D reconstruction.

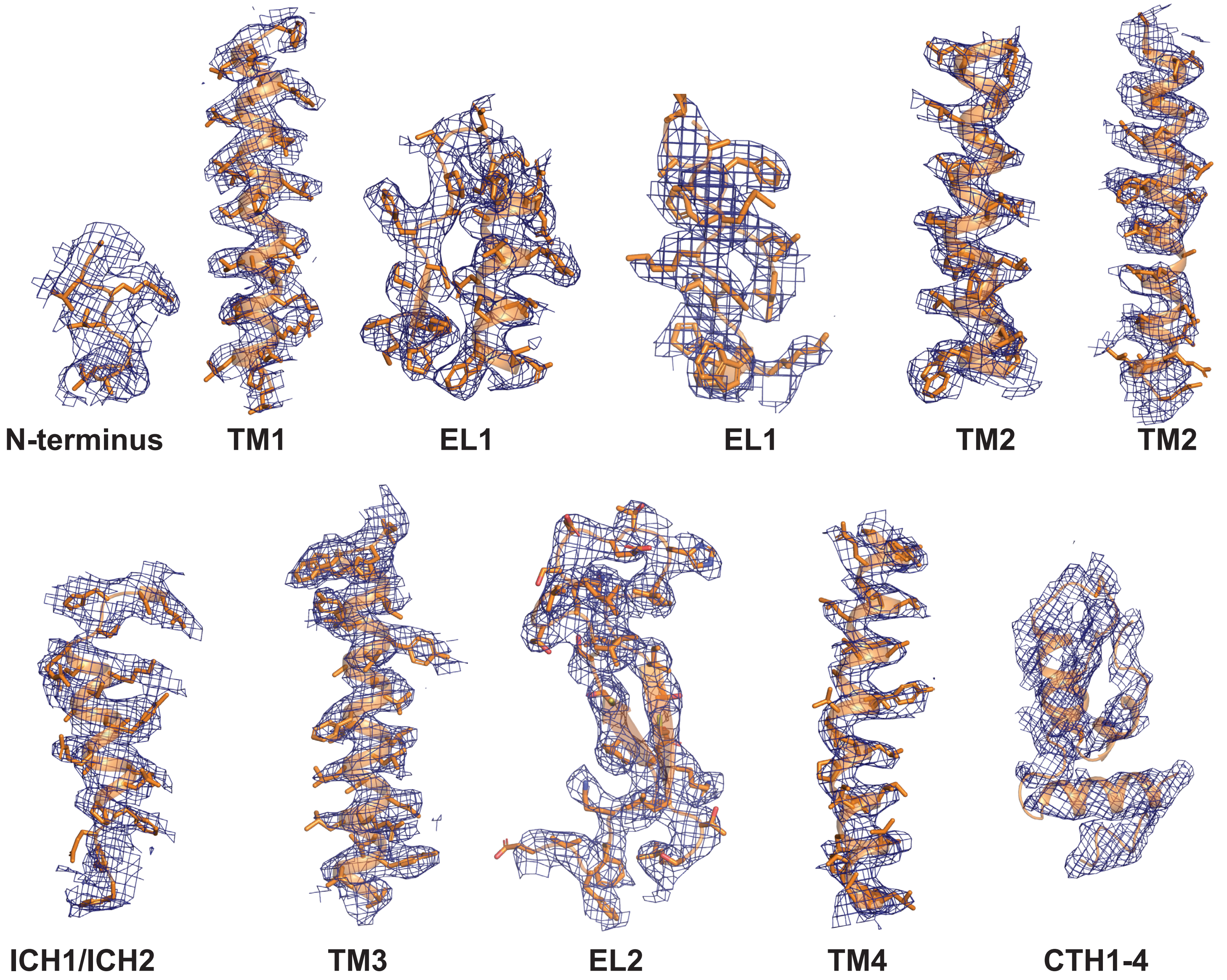

Supplementary Fig. 4: Representative densities for PANX3. The secondary structural elements are labelled according to the topology diagram of PANX3. The structural densities are contoured at 7.5 σ.

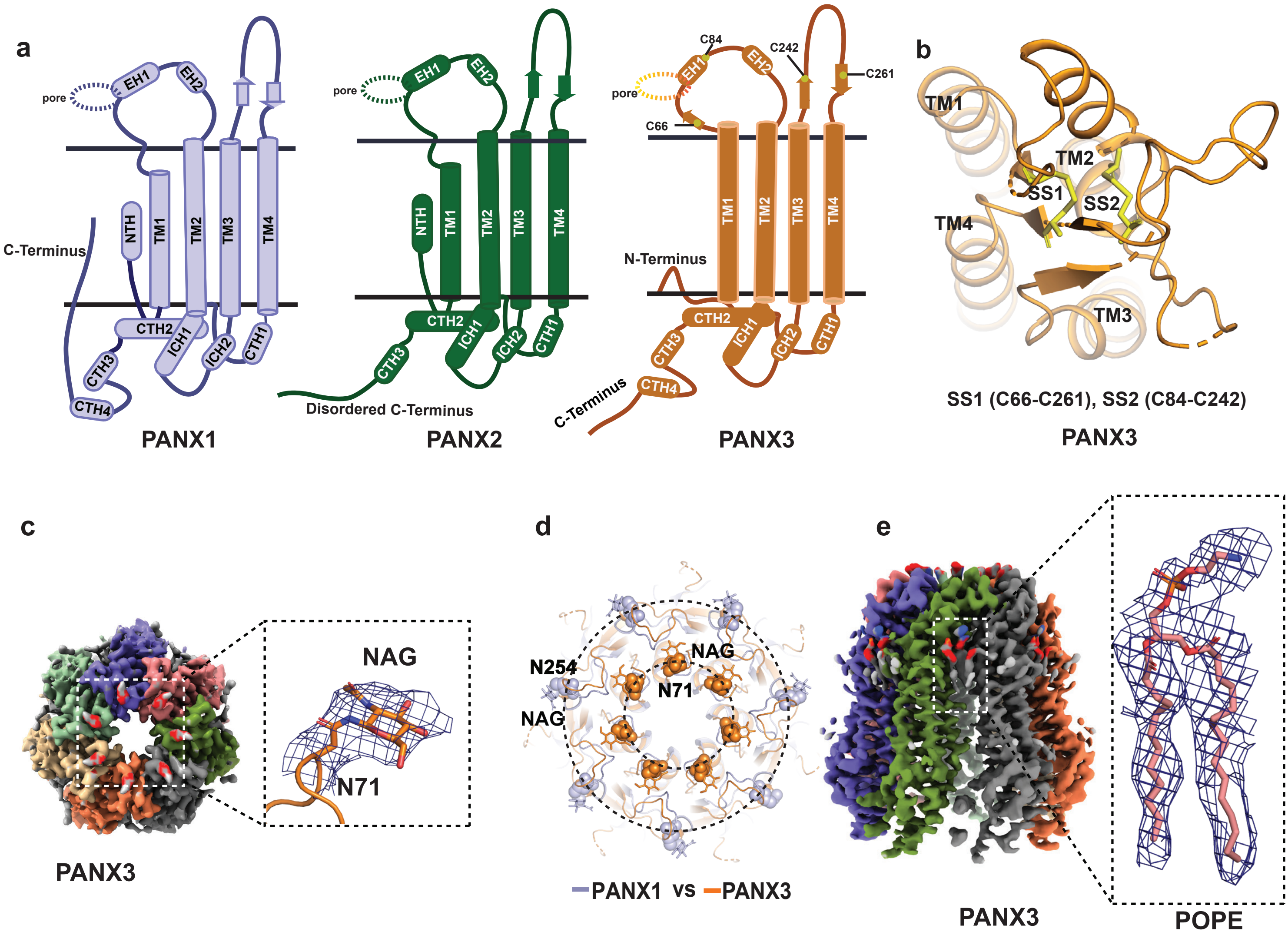

Supplementary Fig. 5: Structural features of PANX isoforms. a, Topology diagram of PANX1(blue), PANX2(green) and PANX3(orange), yellow dots represent the position of cysteines involved in disulfide bond formation in PANX3. TM = Trans-membrane, NTH = N-terminal Helix, ICH = Intracellular helix, CTH = C-terminal helix. **b,** The positions of disulfide bonds in PANX3; SS1(66-261), SS2(84-242) **c-e,** Top view of superposed PANX1 and PANX3 displaying the differences in the position of N-glycosylation in PANX1(blue) and PANX3(orange). Density for NAG, and POPE contoured at 7.5 σ level.

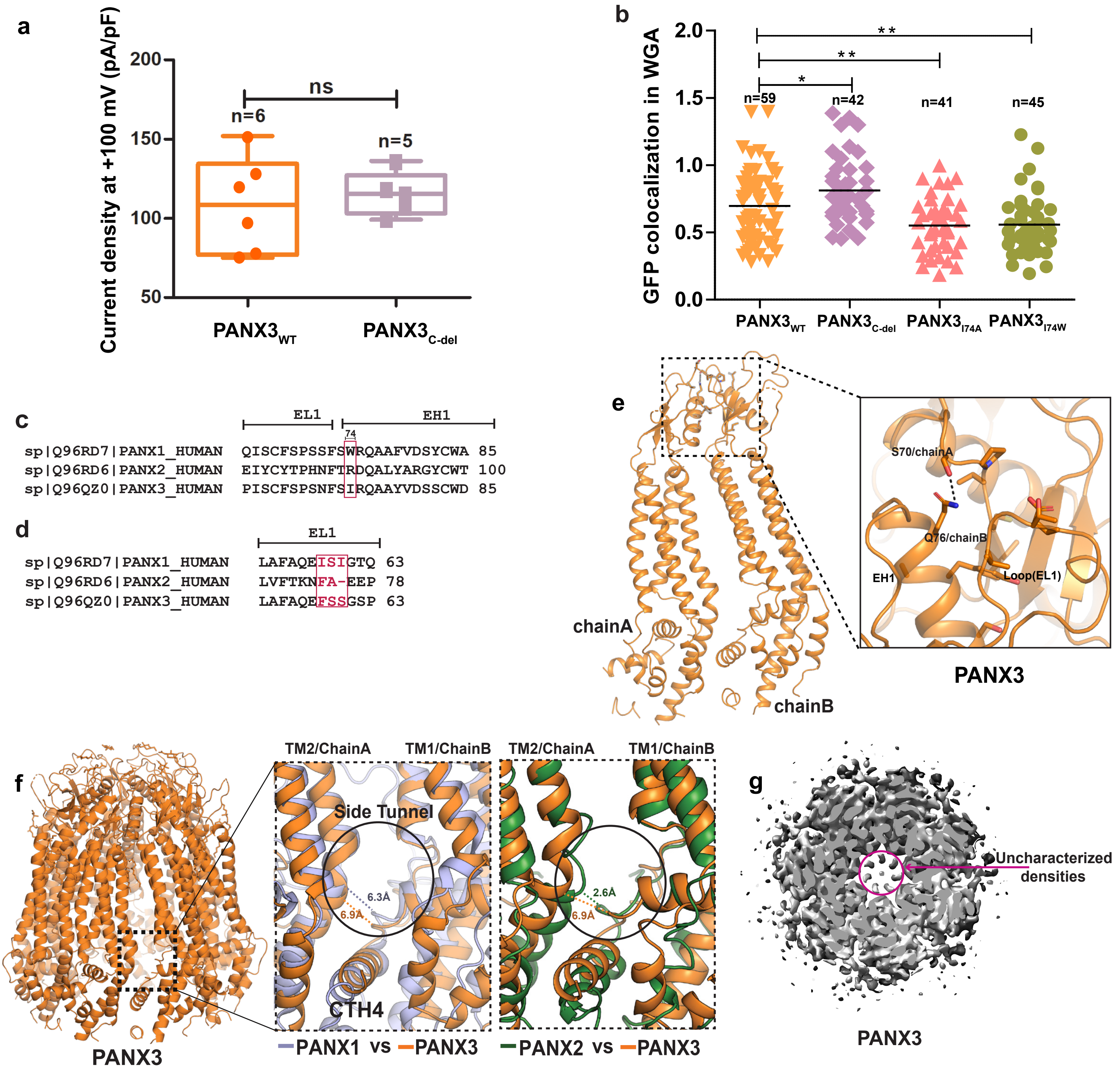

Supplementary Fig. 6: Biochemical analysis of PANX3 and its mutants with a comparative assessment against PANX1 and PANX2 isoforms. **a,** Current density is plotted for the PANX3$_{WT}$(n=6) and PANX1$_{C\text{-}del}$(n=5), n represents the number of cells used for independent recordings; a two-tailed unpaired t-test is used for calculating the significance, n.s., not significant (P value = 0.6595), The whiskers represent minimum and maximum value, the left edge of the box represent 25 % quartile and the right edge represents 75 % quartile, the middle line represents median. Box plot statistics are as follows, for PANX3$_{WT}$, minimum (75.03), 25% percentile (77.00), median (108.6), 75% percentile (134.4), maximum (152.0), for PANX3$_{C\text{-}del}$, minimum (99.20), 25 % percentile (103.01), median (115.3), 75 % percentile (127.1), maximum (136.2), raw traces and the IV curve for the PANX3 mutants are presented in Supplementary Fig. 12. **b,** Surface expression analysis was performed for PANX3 and its mutants. The error bar denotes the standard error of the mean (SEM). 'n' represents the number of cells utilized for the number of cells used for analysis for each mutant, and statistical significance was determined using a two-tailed unpaired t-test, ***p < 0.001; n.s., not significant, PANX3$_{WT}$ vs PANX3$_{C\text{-}del}$ (P value=0.0287), PANX3$_{WT}$ vs PANX3$_{I74A}$ (P value=0.0039), PANX3$_{WT}$ vs PANX3$_{I74W}$ (P value=0.0048), The representative images for the mutants are shown in Supplementary Fig.14 **c,** Multiple sequence alignment showing W74 is not conserved between PANX1, 2 and PANX3 **d,** Multiple sequence alignment exhibiting I58 is not conserved between PANX1, 2 and PANX3 **e,** The interface reveals interactions between the two protomers, specifically between residues S70 and Q76 of chain A and B, respectively. **f,** PANX3 displaying a gap between the protomers; the gap is large enough to accommodate ions and can act as a side tunnel; Inset shows the superposition of PANX1 and PANX3 along with PANX2 and PANX3 vestibule, displaying the position of the side tunnel. **g,** An annulus of seven uncharacterized densities in PANX3 contoured at 7 σ.

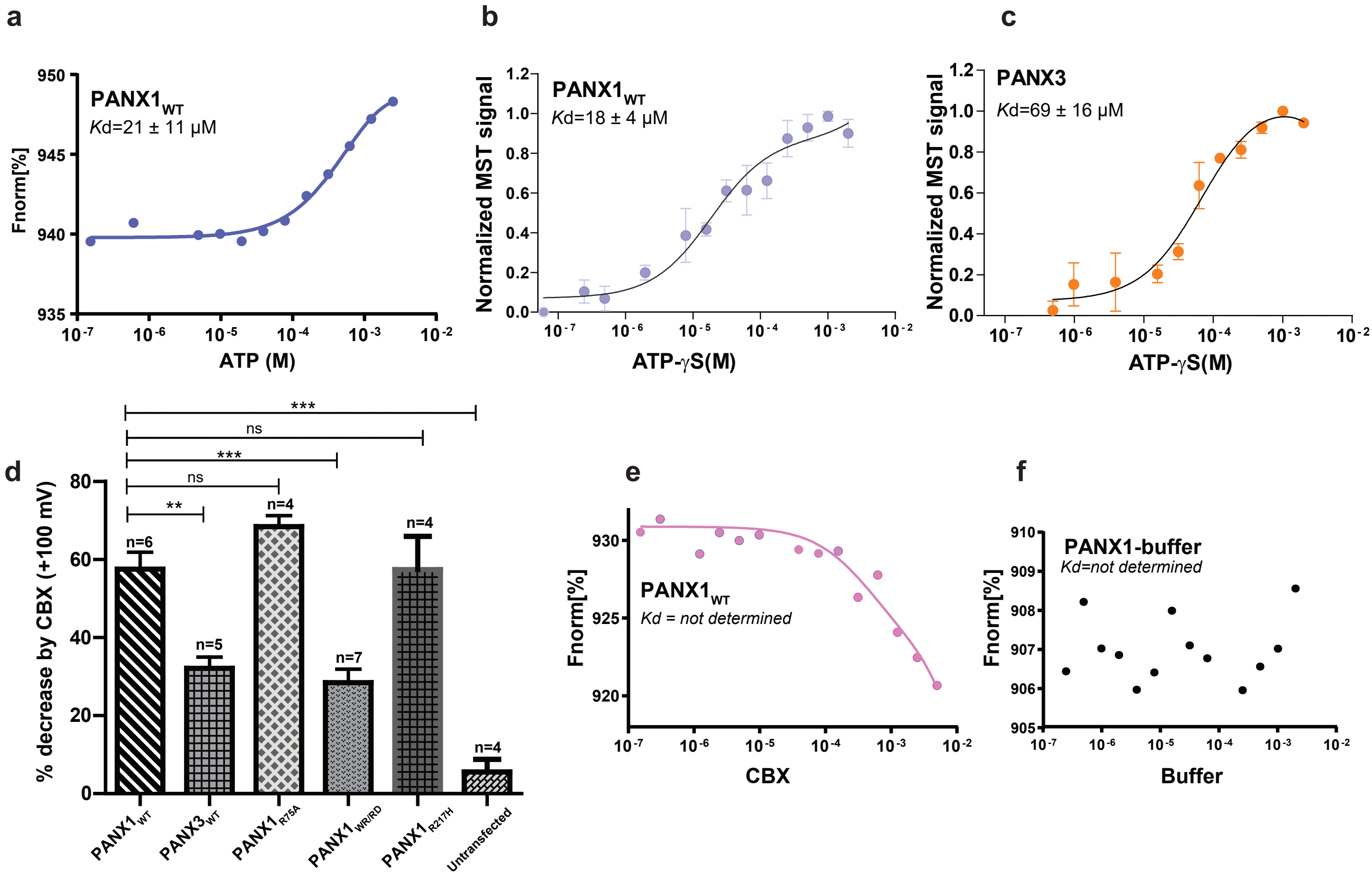

Supplementary Fig. 7: Biochemical analysis of PANX1 and its mutants. **a,** MST binding experiments for PANX1$_{WT}$ with ATP, checked with a single trial, n=1, **b-c** Binding affinity with ATP-γs for PANX1 and PANX3 was determined as 18±4 and 69±16 µM, respectively, n=3, individual experiments, error bar represents S.D. **d,** Percentage inhibition by a PANX inhibitor, CBX plotted at +100mV for wild-type PANX1, 3 and the mutants along with untransfected controls. The number of recordings is mentioned in the graph; error bars represents SEM. A two-tailed unpaired t-test is used for calculating the significance, ***$p < 0.001$; n.s., not significant. PANX1$_{WT}$ vs PANX3 (P value = 0.0012), PANX1$_{WT}$ vs PANX1$_{R75A}$ (P value = 0.1007), PANX1$_{WT}$ vs PANX1$_{WR/RD}$ (P value = 0.0002), PANX1$_{WT}$ vs PANX$_{R217H}$ (P vlaue = 0.9895),PANX1$_{WT}$ vs Untransfected (P value< 0.0001). **e,** CBX binding was not observed with PANX1$_{WT}$ even at a higher (5mM) concentration of CBX, n=1 **f,** Buffer was kept as a control for MST experiments, n=1.

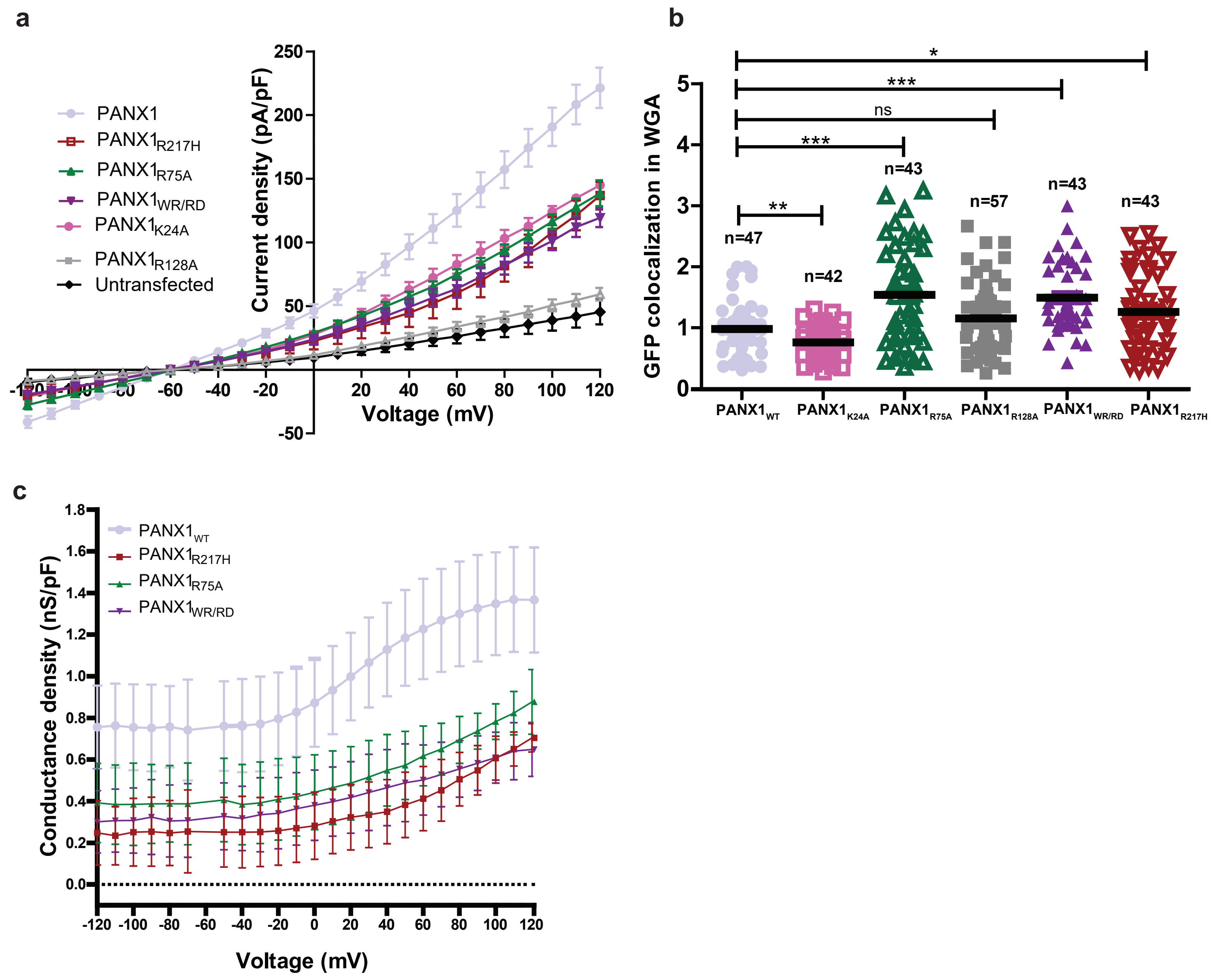

Supplementary Fig. 8: Patch clamp and surface expression analysis for PANX1 and its mutants. a, Current density is plotted for the $PANX1_{WT}$(n=9) and the mutants, $PANX1_{R75A}$(n=10), $PANX1_{R217H}$(n=7), $PANX1_{K24A}$(n=5), $PANX1_{R128A}$(n=7), $PANX1_{WR/RD}$(n=10), untransfected (n=7), the error bar represents SEM **b,** Surface expression analysis for PANX1 and its mutants, n represents the number of the cells used for analysis for each mutant, the error bar represents SEM, $PANX1_{WT}$ vs $PANX1_{R75A}$ (P value=0.0001), $PANX1_{WT}$ vs $PANX1_{WR/RD}$ (P value<0.0001), $PANX1_{WT}$ vs $PANX_{R217H}$ (P value=0.0208), $PANX1_{WT}$ vs $PANX_{K24A}$ (P value=0.0095), $PANX1_{WT}$ vs $PANX_{R128A}$ (P value=0.0929). The representative images for the mutants are presented in Supplementary Figure 14. **c,** Conductance density-Voltage plot for the $PANX1_{WT}$ and the mutants is shown. Each point represents the mean of n = 4-5 individual recordings, and the error bar represents SEM. The number of recordings are as follows, $PANX1_{WT}$(n=5) $PANX1_{R75A}$(n=5), $PANX1_{R217H}$(n=6), $PANX1_{WR/RD}$(n=8).

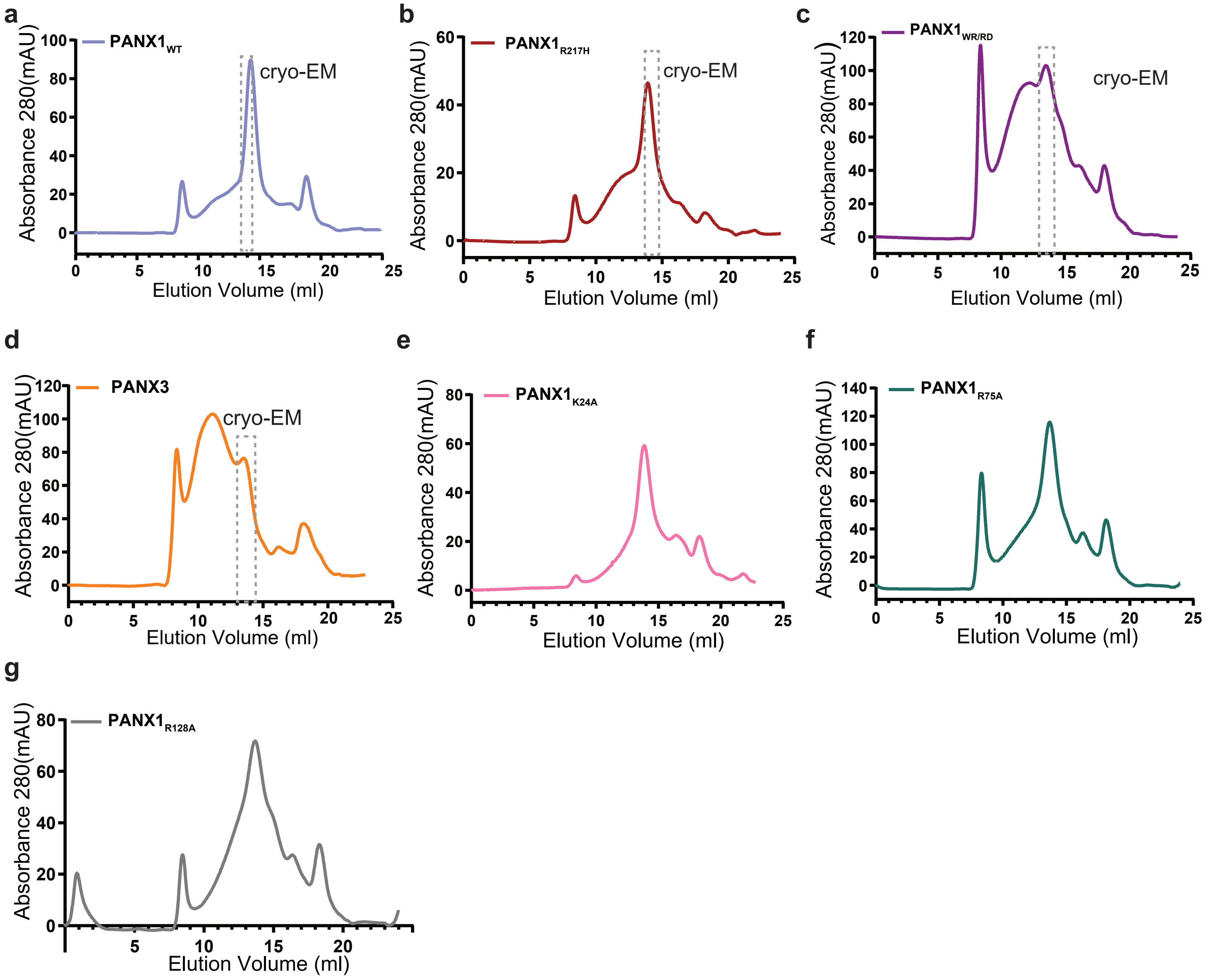

Supplementary Fig. 9: Size exclusion profiles of PANX1 and PANX3 constructs. a, Size exclusion chromatography (SEC) profile for the PANX1$_{WT}$ (blue), the fractions used for cryo-EM grid preparation are marked. **b,** SEC profile for germline mutant PANX1$_{R217H}$ is similar to that of PANX1$_{WT}$. **c,** SEC profile for PANX1$_{WR/RD}$, double mutant, the main fraction is taken for the grid freezing. **d,** SEC profile for PANX3, we observed heterogeneity in the protein, and the protein corresponding to PANX3, labeled as cryo-EM, was used for grid freezing. **e-g,** SEC profiles for PANX1 mutants used for ATP binding studies, PANX-1$_{K24A}$(pink), PANX1$_{R128A}$(gray), and PANX1$_{R75A}$(green). All the proteins were run on a superose6 column.

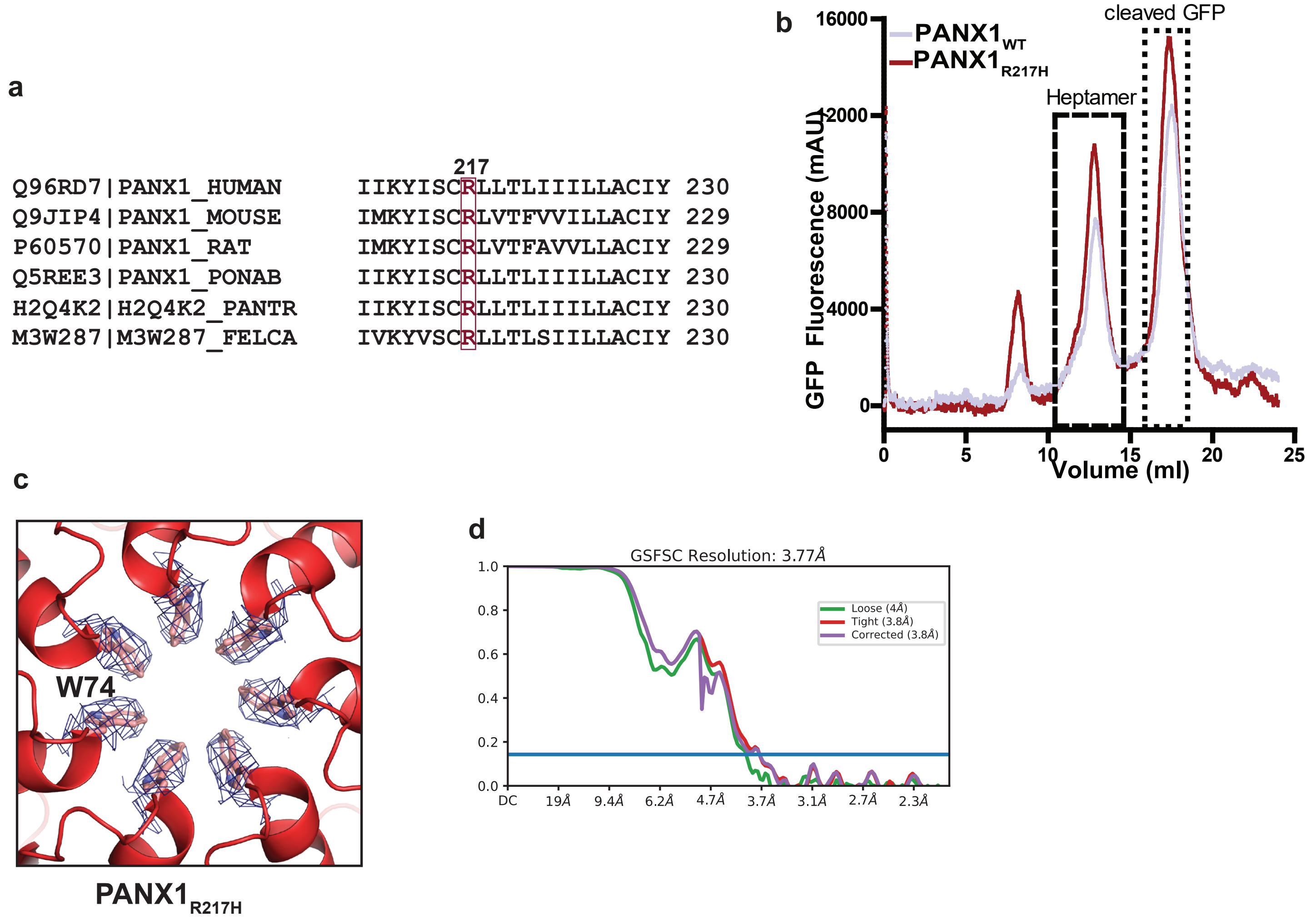

Supplementary Fig. 10: PANX1$_{R217H}$ alters ATP interactions and channel behaviour. a, Multiple sequence alignment of PANX1$_{WT}$ displaying conservation of residues around 217 position. **b,** Fluorescence size exclusion chromatography (FSEC) profile for PANX1$_{WT}$ (blue) on superose6 increase column, the heptamer, and the free GFP is marked on the FSEC profile. **c-d,** The resolution for TMD and ICD was improved by focussed refinement to 3.77 Å, the density for W74 in the improved resolution is displayed at 5.5 σ.

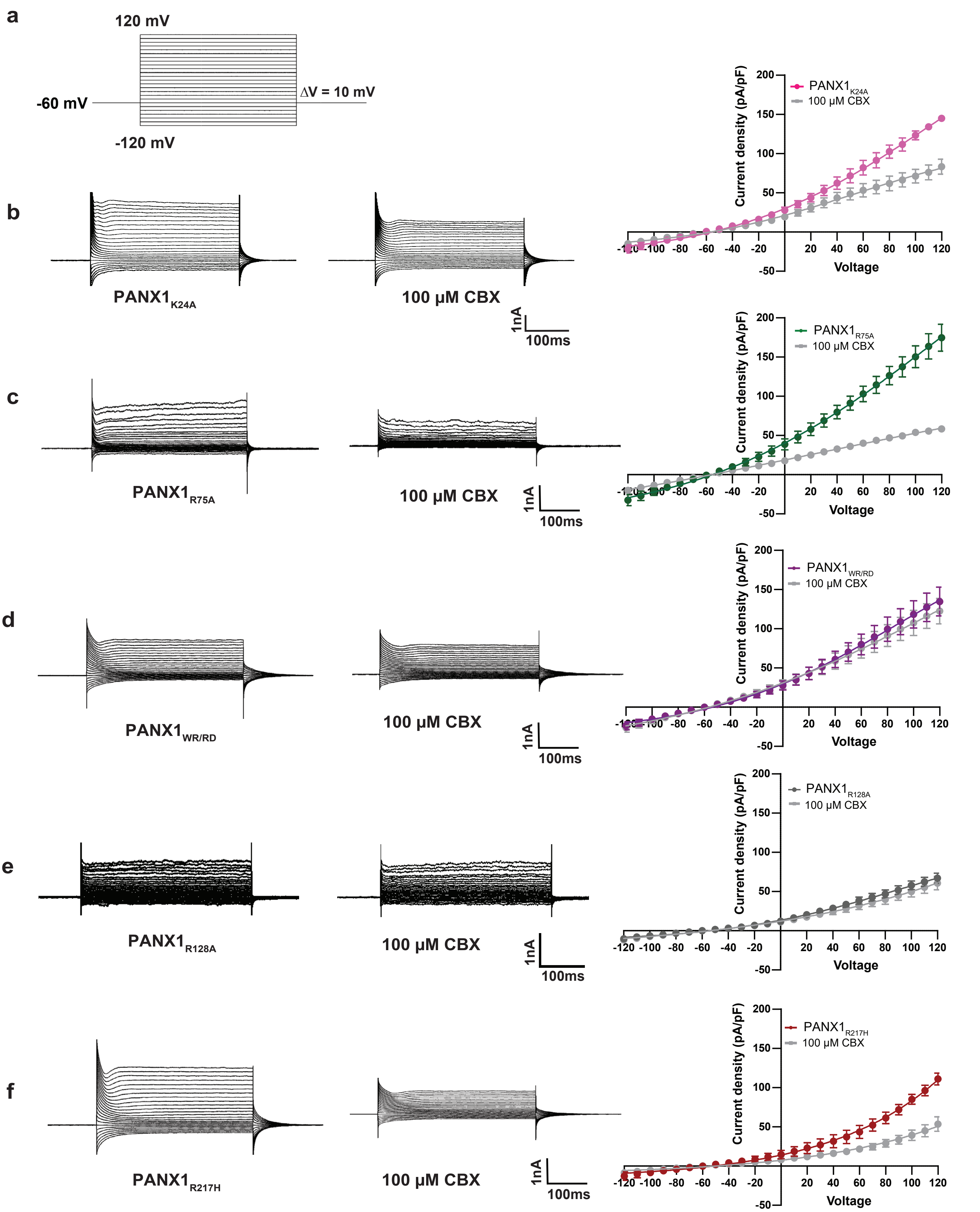

Supplementary Fig. 11: Raw data of patch clamp studies performed with PANX1. a, The protocol used for patch clamp is shown; the voltage steps of 10/20mV were applied from -120 to +120, mock recordings(untransfected HEK293 cells) were done prior to the $PANX1_{WT}$ and mutant experiments; **b-f**, Representative raw traces along with the current density-voltage plot for whole-cell current for HEK cells expressing $PANX1_{WT}$ and the mutants are displayed, 100µM CBX was applied to study the effect of the inhibitor on PANX channels. Each point represents the mean of n = 4-6 individual recordings, and the error bar represents SEM. The number of recordings are as follows, $PANX1_{K24A}$(n=4) $PANX1_{R75A}$(n=4), $PANX1_{WR/RD}$(n=6), $PANX1_{R128A}$(n=4), $PANX1_{R217H}$(n=4)

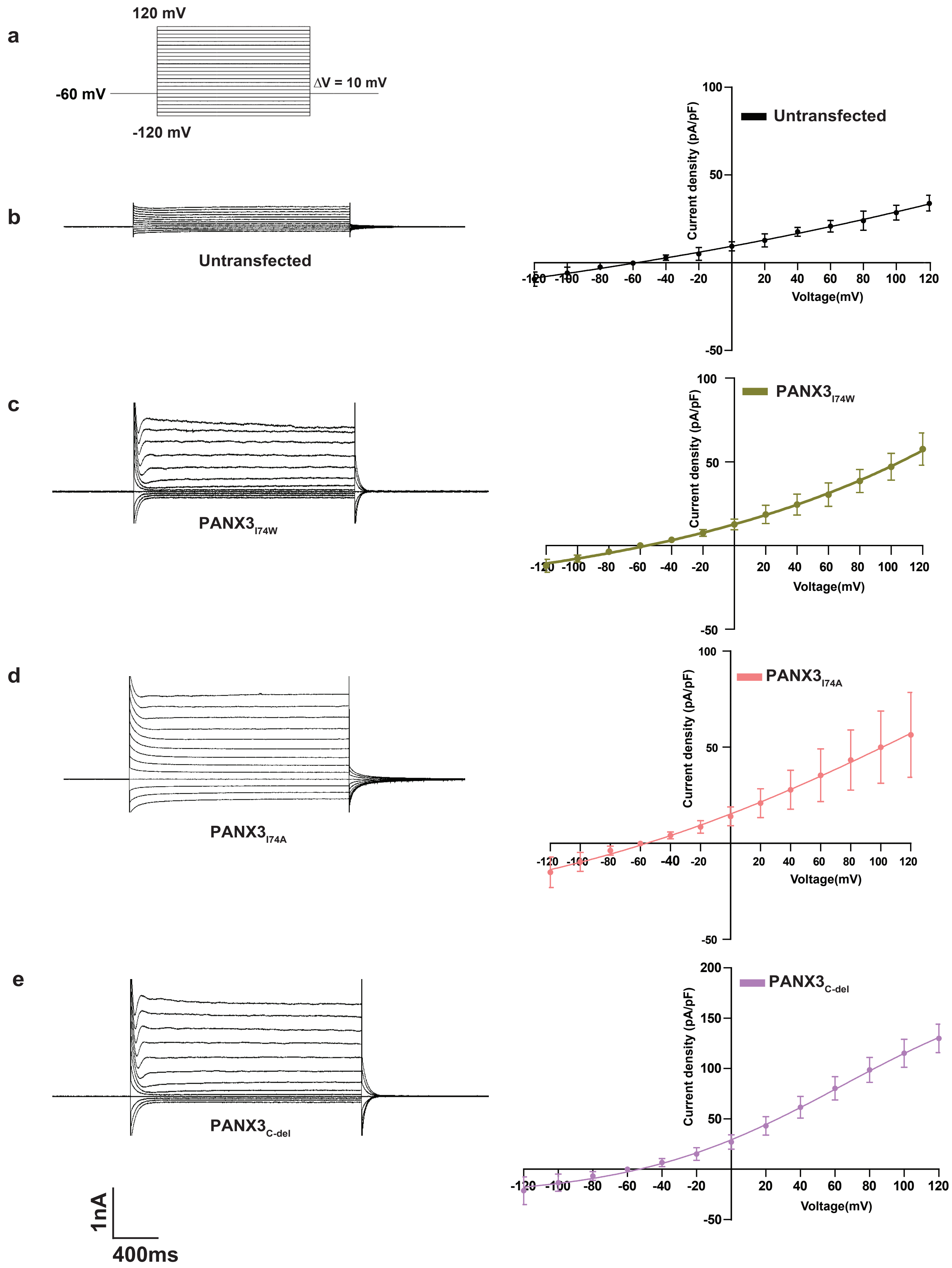

Supplementary Fig. 12: Raw data from patch clamp studies involving PANX3. a,The patch clamp protocol utilized voltage steps of 10/20mV across the range of -120 to +120. **b,** Mock recordings were initially conducted on untransfected HEK293 cells before proceeding to experiments involving PANX3$_{WT}$ and mutants. **c-e**, Representative raw traces along with the current density-voltage plot for whole-cell current for HEK cells expressing PANX3 mutants are displayed. The number of recordings are as follows, PANX3$_{I74A}$ (n=7), PANX1$_{I74W}$ (n=4), PANX1$_{c\text{-}del}$ (n=5), Untransfected (n=5). Error bar represents SEM

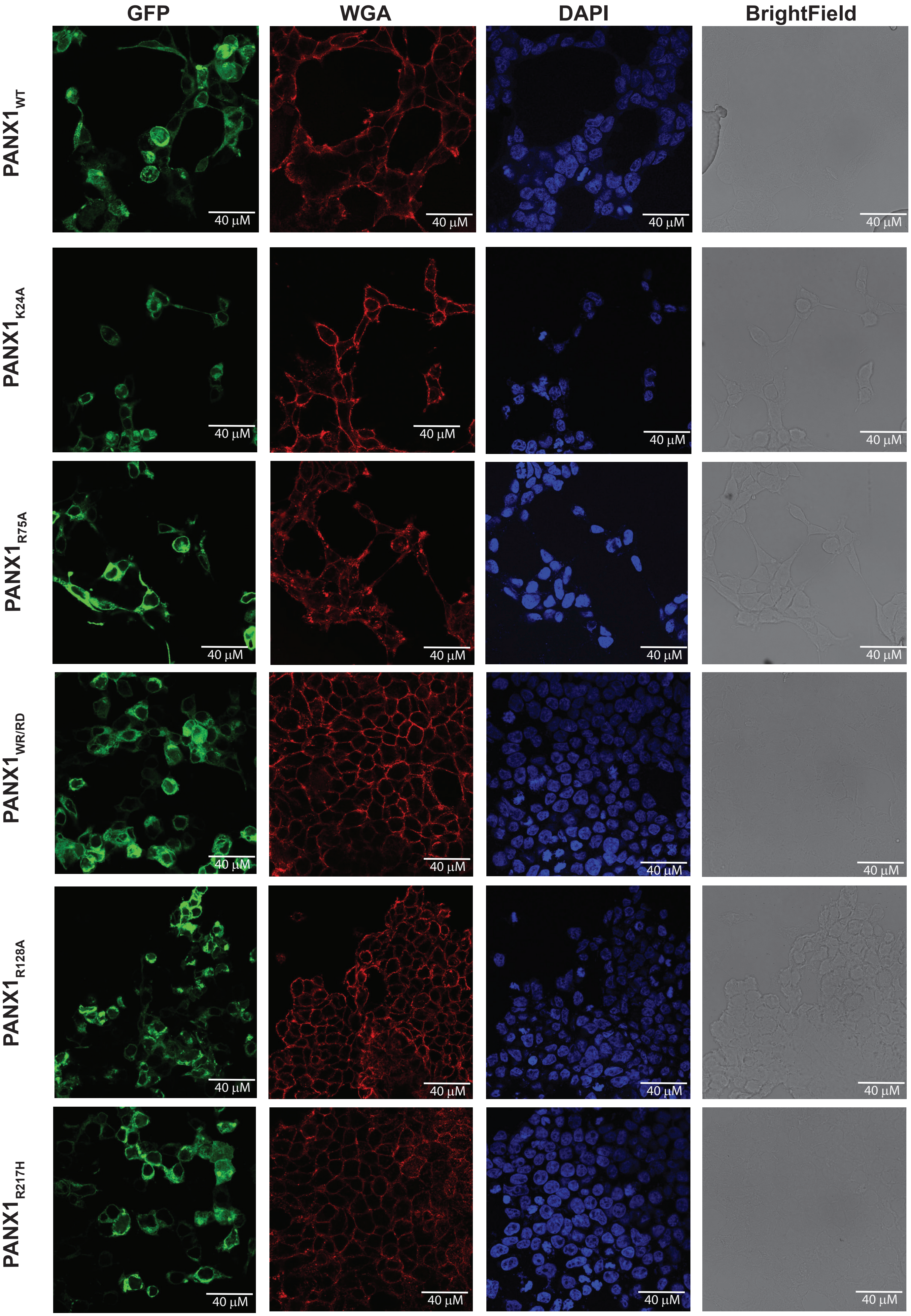

Supplementary Fig. 13: Surface expression of wild-type PANX1 and the mutants. The Surface expression of wild-type PANX1 and the mutants was evaluated through immunofluorescence staining. Representative images of immunofluorescent staining for wild-type human PANX1 and mutants are shown. GFP = Green Fluorescence Protein (Green), WGA = Wheat Germ Agglutinin (Red), DAPI = 4',6-diamidino-2-phenylindole (blue).

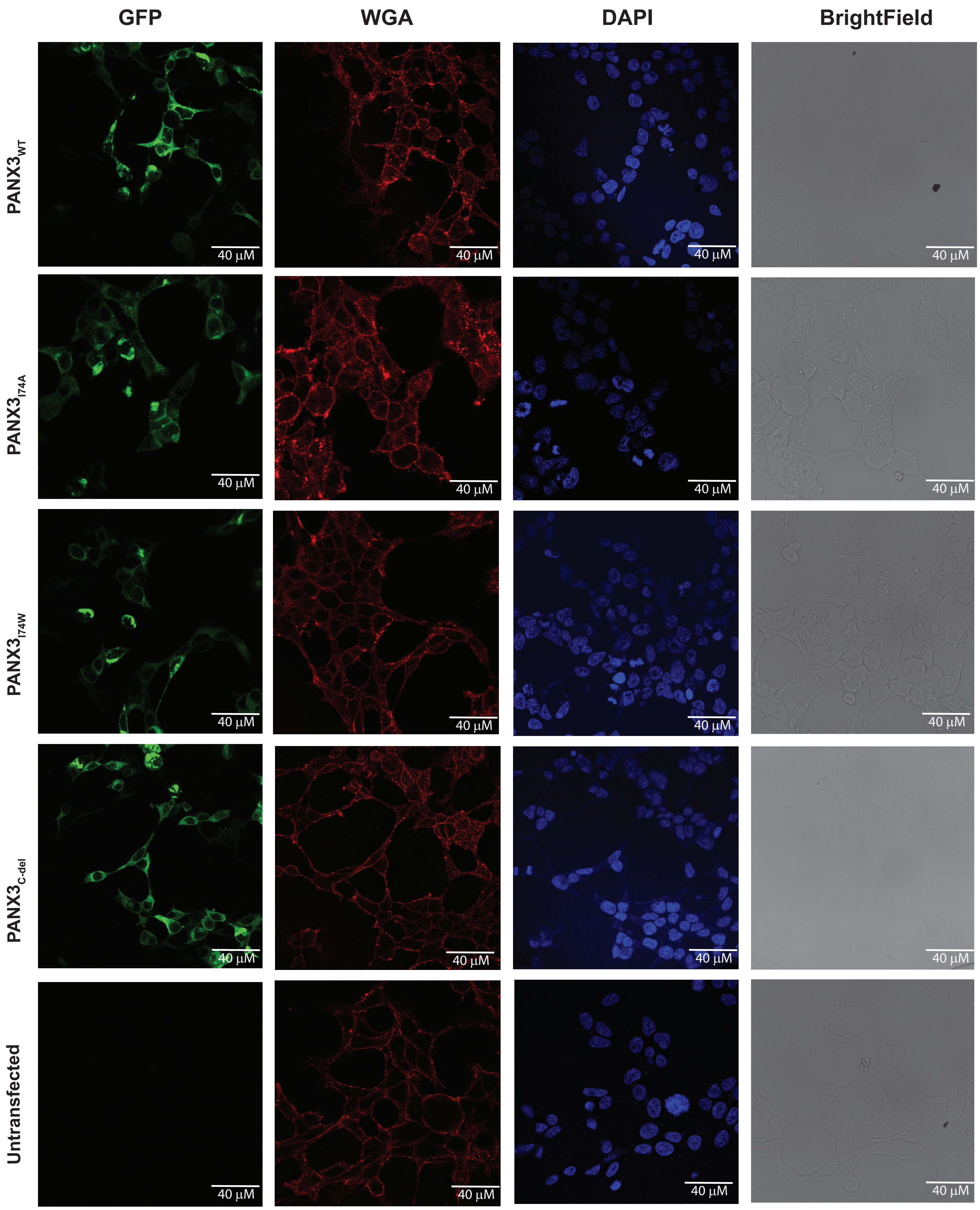

Supplementary Fig. 14: Surface expression of wild-type PANX3 and the mutants. The surface expression of wild-type PANX3 and its mutants was assessed using immunofluorescence staining. Representative images of immunofluorescent staining for wild-type human PANX3 and mutants are presented, with GFP indicating Green Fluorescent Protein (green), WGA denoting Wheat Germ Agglutinin (red), and DAPI representing 4',6-diamidino-2-phenylindole (blue).

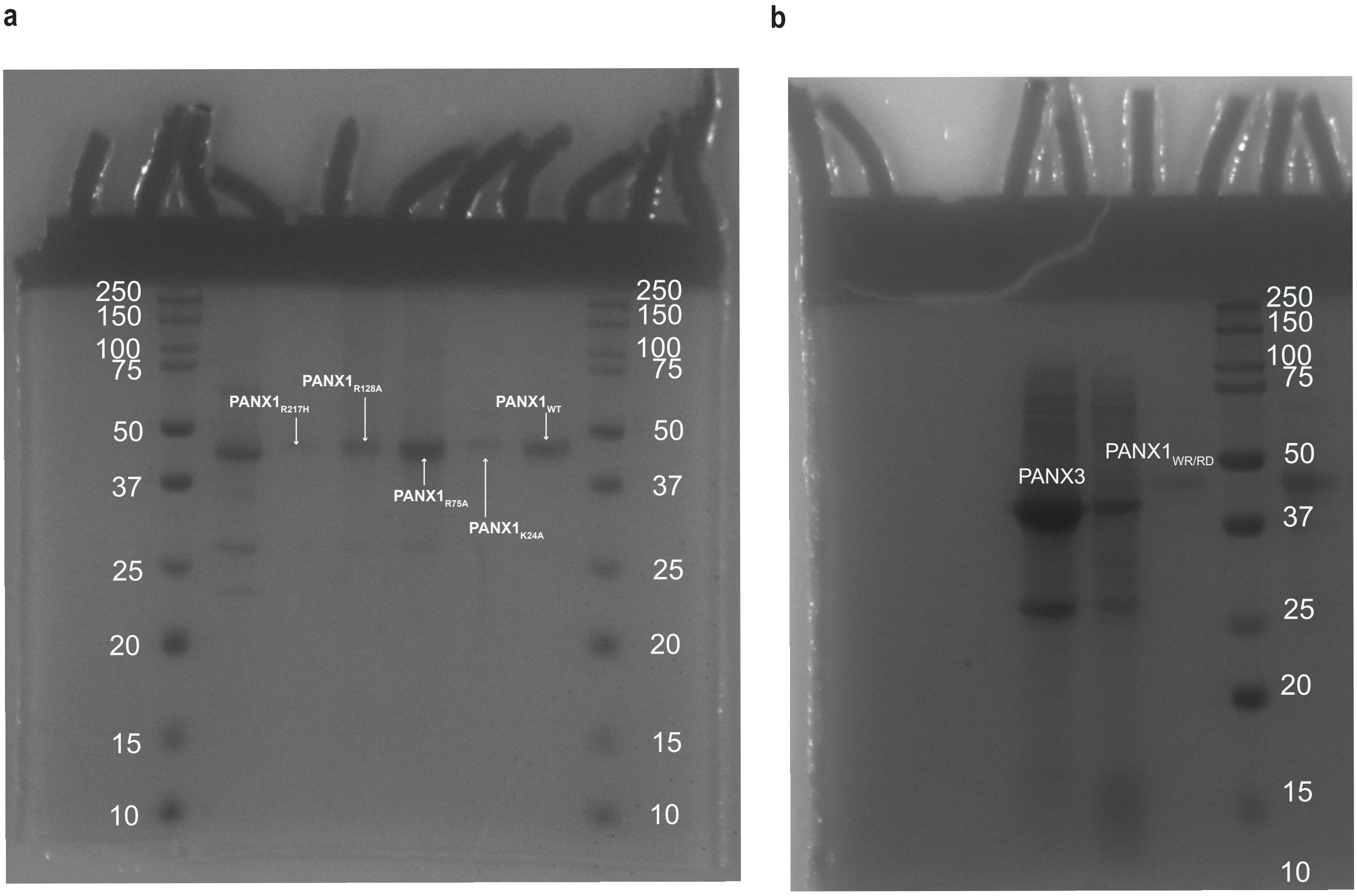

Supplementary Fig. 15: SDS-PAGE profile for the constructs mentioned in the study, a, for PANX1$_{WT}$ and the mutants (from right to left, PANX1$_{K24A}$, PANX1$_{R75A}$, PANX1$_{R128A}$, PANX1$_{R217H}$), **b,** for PANX3$_{WT}$ and PANX1 pore mutant, PANX1$_{WR/RD}$.

I74A_PANX3_FP	AGTAACTTCAGCGCCCGGCAGGCAGC
I74W_PANX3_FP	AGTAACTTCAGCTGGCGGCAGGCAGC
C-del_PANX3_RP	GATTGGAAGTACAGGTTTTCCAATAAAGTCATAAAATCAA
K24A_PANX1_FP	ACGGAGCCCGCCTTCAAGGGGCTG
R29A_PANX1_FP	AAGGGGCTGGCCCTGGAGCTGGC
K36A_PANX1_FP	TGGCTGTGGACGCCATGGTCACGT
R128A_PANX1_FP	GCTGTTCTGGGCCTTCGCAGCTGCT
R75A_PANX1_FP	TTCTTTCTCCTGGGCTCAGGCTGCCTTTG
R217H_PANX1_FP	TACATTAGCTGCCATCTGCTGACACTC
WR/RD_PANX1_FP	TCCAAGTTCTTTCTCCCGTGATCAGGCTGCCTTTGTGGA
BAMS FP	CTGATAGGCACCTATTGGTCTTAC
BAMS RP	ACCATAAGAATTGATACAACGAGGAAAATGCG

Supplementary Table 1: List of Primers used in the study along with their sequences.
