# Supplementary material for: Cryo-EM structures of pannexin 1 and 3 reveal differences among pannexin isoforms
Source: Nat Commun. 2024 Apr 5;15:2942. doi: 10.1038/s41467-024-47142-6 (PMC10997603; doi:10.1038/s41467-024-47142-6)
Supplement: Supplementary file 3 — Description of Additional Supplementary Files [file 41467_2024_47142_MOESM3_ESM.pdf]

File name: Supplementary Movie 1

Description: The movement of W74 by 80° at the $\chi 2$ torsion angle leads to reduction in pore size in PANX1 congenital mutant.
